# Bili Inhibits Wnt/β-Catenin Signaling by Regulating the Recruitment of Axin to LRP6

**DOI:** 10.1371/journal.pone.0006129

**Published:** 2009-07-02

**Authors:** Lorna S. Kategaya, Binita Changkakoty, Travis Biechele, William H. Conrad, Ajamete Kaykas, Ramanuj DasGupta, Randall T. Moon

**Affiliations:** 1 Department of Pharmacology, Howard Hughes Medical Institute (HHMI), University of Washington School of Medicine and Institute for Stem Cell and Regenerative Medicine, University of Washington School of Medicine, Seattle, Washington, United States of America; 2 Department of Pharmacology and Cancer Institute, New York University School of Medicine, New York, New York, United States of America; Max Planck Institute of Molecular Cell Biology and Genetics, Germany

## Abstract

**Background:**

Insights into how the Frizzled/LRP6 receptor complex receives, transduces and terminates Wnt signals will enhance our understanding of the control of the Wnt/ß-catenin pathway.

**Methodology/Principal Findings:**

In pursuit of such insights, we performed a genome-wide RNAi screen in *Drosophila* cells expressing an activated form of LRP6 and a β-catenin-responsive reporter. This screen resulted in the identification of Bili, a Band4.1-domain containing protein, as a negative regulator of Wnt/β-catenin signaling. We found that the expression of Bili in *Drosophila* embryos and larval imaginal discs significantly overlaps with the expression of Wingless (Wg), the *Drosophila* Wnt ortholog, which is consistent with a potential function for Bili in the Wg pathway. We then tested the functions of Bili in both invertebrate and vertebrate animal model systems. Loss-of-function studies in *Drosophila* and zebrafish embryos, as well as human cultured cells, demonstrate that Bili is an evolutionarily conserved antagonist of Wnt/β-catenin signaling. Mechanistically, we found that Bili exerts its antagonistic effects by inhibiting the recruitment of AXIN to LRP6 required during pathway activation.

**Conclusions:**

These studies identify Bili as an evolutionarily conserved negative regulator of the Wnt/β-catenin pathway.

## Introduction

Wnt signaling through β-catenin regulates the expression of genes involved in cell proliferation and cell fate during development, in adult homoeostasis, and in diverse diseases [1]. In the absence of a Wnt ligand, steady-state levels of β-catenin are maintained at a low level by a ‘degradation complex’ that promotes the phosphorylation, ubiquitination, and proteosomal degradation of β-catenin [2]. The binding of Wnt ligands to the transmembrane FZD (frizzled) and LRP5/6 (low-density-lipoprotein-related protein 5/6) co-receptors triggers an inhibition of the degradation complex resulting in the stabilization and nuclear accumulation of β-catenin, where it binds to transcription factors of the TCF/LEF family and regulates target gene expression.

Signaling events immediately following Wnt binding to the receptor complex are poorly understood. Previous studies have shown that in the presence of Wnt ligand, Dishevelled (DVL) facilitates phosphorylation of the cytoplasmic tail of LRP5/6 [3] by Casein Kinase 1γ (CSNK1G1) and Glycogen Synthase Kinase-3β (GSK3B) [4], [5]. Phosphorylation of LRP5/6 is followed by the recruitment of AXIN away from the degradation complex. As AXIN is likely a rate-limiting scaffold protein required for the degradation of β-catenin [6], the relocation of AXIN to the membrane away from the degradation complex likely promotes stabilization of β-catenin [7].

We performed a genome-wide RNAi screen in *Drosophila* cells to identify genes that regulate LRP6 mediated activation of a β-catenin reporter. Here we describe Bili (Band4.1 inhibitor LRP interactor), a previously uncharacterized FERM domain containing protein. Analyses of Bili function in *Drosophila*, zebrafish, and cultured human cells support the conclusion that Bili is an evolutionarily conserved antagonist of β-catenin signaling. Mechanistically, we show that Bili regulates the recruitment of AXIN to LRP6.

## Results

### RNAi screen in Drosophila identifies Bili as a negative regulator of Wg signaling

*Drosophila* mutagenesis screens have resulted in the identification of several core Wg signaling proteins [8]. The potential for discovering additional contextually relevant molecular players in the pathway is enhanced by robust read-outs such as transcriptional reporter assays coupled with genome-wide RNAi libraries [9]. Our goal was to identify new proteins that regulate Wnt/β-catenin signaling at the level of the receptor complex. While Wnt and Frizzled proteins can signal independent of β-catenin, signaling through β-catenin is dependent on LRP5/6 co-receptors [7]. Therefore, we modified a *Drosophila* dsRNA screen [10] by using a constitutively active LRP6 mutant (ΔNLRP6) [11] to activate a fly-optimized β-catenin luciferase reporter, dTF12 [10]. In a high-throughput RNAi screen, *clone 8* (*cl8*) *Drosophila* cells were transfected in a 384-well plate format with individual dsRNAs, dTF12, ΔNLRP6 and a *Renilla* luciferase control for cell viability and transfection efficiency. The cells were incubated for four days to allow knockdown of target RNAs and normalized reporter luminescence was then measured as an indicator of Wnt pathway activity. Approximately 0.5% of the library (∼100 dsRNAs) modified the activation of the reporter by ΔNLRP6 in a manner validated by low throughput confirmation of the primary screen. dsRNA targeting eighteen genes with known human orthologs increased reporter activation two-fold or greater (Table S1). Seven of the eighteen amplicons that had potential off-target effects according to the publicly available *Drosophila* RNAi Screening Center (DRSC, flyrnai.org) database were not pursued further.

### Bili is a conserved FERM-domain protein

Of the 11 remaining candidate negative regulators, we chose to focus on CG11848 (dBili) due to its predicted plasma membrane localization and the specificity of its effect on the Wg pathway. More specifically, of the 12 other screens in the DRSC database, dBili was identified only as a weak hit in a MAPK kinase screen (Friedman et. al., 2007). Bili, annotated as CG11848 (fly) and *FRMD8*/FKSG44 (human), also has orthologs in worms, zebrafish and mice (Fig. S1A). In human tissue samples, we found Bili mRNA expression highest in heart and spleen (Fig. S1B). Bili is a predicted 464 amino acid protein with a Band4.1/FERM domain in its N-terminus (amino-acids, 26–272) (Fig. S1C). FERM domains (4.1 Ezrin Radixin Meosin) are highly conserved protein domains important for directing plasma membrane association and, in some cases, linking integral membrane proteins to the actin cytoskeleton and modulating signaling [12]. Given that no prior Band4.1 domain proteins have been implicated in Wnt signaling, we focused on Bili to determine whether it functioned in a unique capacity in the Wnt/β-catenin pathway.

### Bili dsRNA validates in low throughput assays

**Figure 1:**
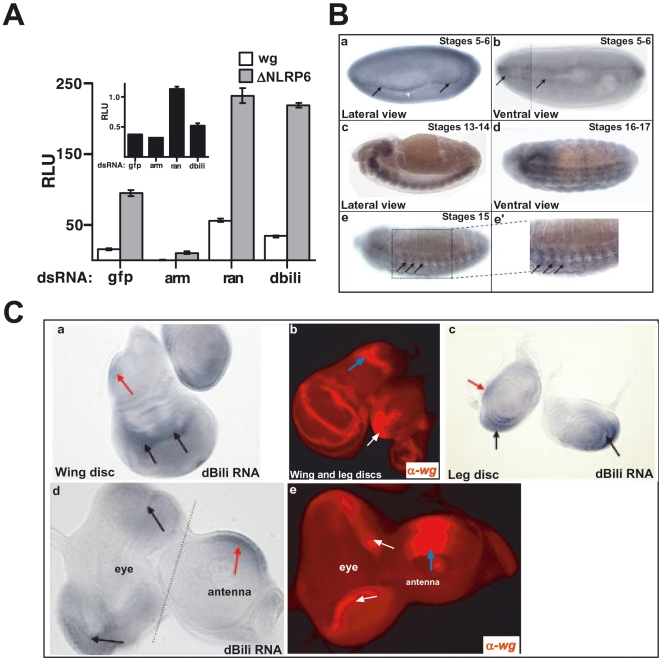
dBili (cg11848) is a negative regulator of Wg signaling. (A) Secondary reporter assay in clone 8 cells showing gfp, armadillo (arm), ran and dBili dsRNA effects on wingless (wg) (white bars) and ΔNLRP6 (gray bars) activation of β-catenin reporter, dTF12. Individual dsRNA effects on reporter activity in the absence of wg or ΔNLRP6 (inset). (B) Expression pattern of dBili mRNA by in situ hybridization. Embryos are oriented with anterior on the left and posterior on the right. (a & b) Stages 5–6 showing dBili expression in the neuroectoderm and cells adjacent to the ventral midline (black arrows) with high levels of expression in cells adjacent to the ventral furrow (white arrowhead). (c–e). At stages 13–17 the expression of dBili in the developing central nervous system (panel c & e) and the ventral epidermis in stripes (e & e'). At the end of embryogenesis (stage 16/17), the majority of dBili expression is restricted to the CNS (panel d). (C) (a & b) Wing imaginal discs. Panel a, shows dBili expression in the wing pouch (black arows) and in the notum/hinge area (red arrow). Panel b, staining for Wg protein in wing pouch and notum/hinge (blue arrow). (b & c) Leg imaginal discs. Panel c shows dBili staining in the ventral wedge (black arrows), identical to Wg expression (white arrow, panel b) (d & e) Expression in the eye disc of dBili (black arrows, d) and Wg (white arrows, e). Expression in the antenna disc of dBili (d, red arrow) and Wg (e, blue arrow).

dsRNA targeting either dBili or Ran, a previously identified negative regulator of Wg signaling [13], enhanced the activation of dTF12 by either ΔNLRP6 or Wg (Fig. 1A). As expected, dBili dsRNA decreased endogenous dBili transcripts compared to control dsRNA (Fig. S3A). A second independent dsRNA targeting dBili, dsBili_2, also decreased dBili transcript levels and enhanced ΔNLRP6-activated reporter activity (Fig. S3A and B). Additionally, overexpressing dBili in S2R+ or Clone8 Drosophila cell lines inhibits *Wg*-dependent reporter activation (Fig. S3C and D). These data support the conclusion that dBili is a novel negative regulator of Wg signaling.

### Bili is coexpressed with Wg during Drosophila development

If dBili is a component of the Wg pathway in *Drosophila*, then it should be co-expressed with other genes involved in Wg signaling. To test this hypothesis we performed *in situ* hybridization of dBili in *Drosophila* embryos (Fig. 1B) and larval imaginal discs (Fig. 1C). At early stages (stages 5–6) dBili RNA is uniformly expressed in the early embryonic ectoderm at low levels (data not shown), and more highly expressed in the neuroectoderm (Fig. 1B, a and b). Additionally, dBili is expressed in cells adjacent to the ventral midline just prior to initiation of the invaginating ventral furrow (black arrows, Fig. 1B, a and b). Interestingly, at later stages (stages 13–17), the expression of dBili is evident in the developing central nervous system (CNS, Fig. 1B, c and e) and the ventral epidermis in stripes (black arrows, Fig. 1B, e and e') similar to the expression of Wg (Riggleman et al. 1990). At the end of embryogenesis (stage 16/17), the majority of dBili expression is restricted to the CNS (Fig. 1B, d).

dBili and Wg expression patterns also overlap in wing, leg, and the eye-antennal imaginal discs in third instar larvae (Fig 1C). In the wing imaginal disc, dBili is expressed broadly in the wing pouch (black arrows, Fig. 1C, panel a) and in the notum (red arrow, Fig. 1C, panel a). The expression of dBili in the notum and that of Wingless (Wg) protein (blue arrow, Fig. 1C, panel b) appears to be non-overlapping, however they do abut each other. In the leg disc, dBili is expressed in a small discrete domain in the posterior leg disc (red arrow, Fig. 1C, panel c) as well as in the ventral wedge (black arrows, Fig. 1C, panel c) in a pattern slightly broader yet nearly identical to Wg expression (white arrow, Fig. 1C, panel b). In both dorsal and ventral compartments of the eye disc, dBili expression (black arrows, Fig. 1C, panel d) partially overlaps with the lateral edge expression of Wg (white arrows, Fig. 1C, panel e). In the antennal disc, dBili is expressed as a dorsal wedge (red arrow, Fig. 1C, panel d) which also overlaps with that of Wg (blue arrow, Fig. 1C, panel e). We conclude that the overlapping and adjacent expression of dBili and Wg are consistent with dBili functioning in the *Wg* pathway.

### dBili negatively regulates Wg signaling during Drosophila embryogenesis

We next investigated the function of dBili during *Drosophila* embryogenesis. Wg has a well-established role in patterning the ventral epidermis and has been shown to be involved in cell fate determination [14], [15]. Epidermal cells with active Wg signaling secrete naked-cuticle, whereas cells lacking Wg signaling activity secrete denticle belts. If dBili is a negative regulator of Wg signaling, reduced Bili function should resemble Wg gain of function phenotypes including ectopic naked cuticle.

**Figure 2:**
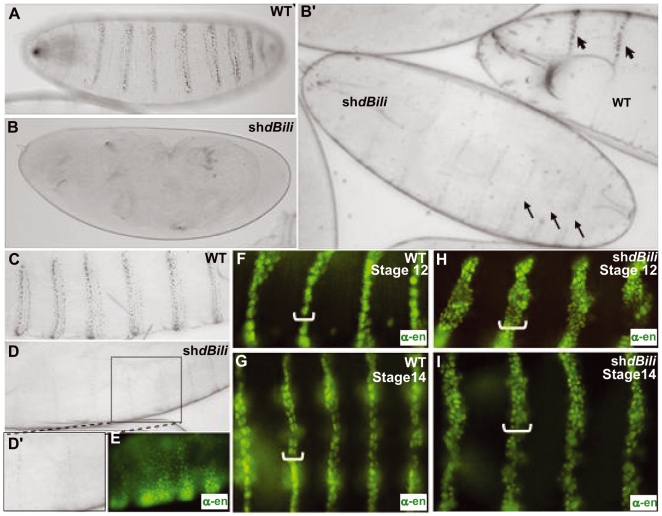
Expansion of wg signaling activity in embryos (ventral view) with diminished levels of dBili. (A) Cuticle preparation of WT control embryo displaying wild type patterning of the embryonic epidermis with uniformly spaced denticle belts. (B) Cuticle preparation of embryo expressing shRNA targeting dBili (shdBili). (B') WT and shdBili expressing embryos showing a weaker phenotype (black arrows). (C & D) High magnification view of A & B, respectively. D' & E show boxed region in D. (E–I) Engrailed (En) protein expression. (F & G) Expression of engrailed in WT embryos is restricted to one or two cell-layers in the epidermis during early stage 12 (germband retraction) and in the ventral epidermis at stage 14 (onset of head involution and dorsal closure). (H & I) En expression in shdBili stage-matched embryos (compare with F & G).

dBili levels were reduced during embryogenesis by expressing a short-hairpin RNA (shdBili) in the paired domain (prdGAL4>UAS-shdBili). Quantitative real time-PCR (qRT-PCR) analysis following expression of dBili shRNA under the control of prd-GAL4 in the *Drosophila* embryo revealed >75% knockdown of dBili mRNA levels compared to prd-GAL4 control embryos (Fig. S2A). As a control for the specificity of shdBili we also quantified levels of *armadillo* mRNA and found no change (Fig. S2A). Importantly, the expression of shdBili led to the expansion of Wg signaling activity as demonstrated by the secretion of mostly naked cuticle with very few cells secreting cuticle with trichomes (denticles) (Fig. 2A and 2B). Furthermore, driving dBili shRNA expression with *even skipped* stripe 3/7-GAL4, disrupted denticle stripe 2/3 (Fig. S2B). Thus, depleting dBili in the *Drosophila* embryo phenocopies hyperactivated Wg signaling, suggesting that dBili is a negative regulator of the Wg pathway in *Drosophila* embryos.

Next we assessed the molecular consequences of depleting dBili in the paired domain. During embryonic segmentation, Wg activates and maintains the expression of *engrailed* (En) and defines para-segment boundaries [16]. Consistent with Wg gain-of- function, immunohistochemistry for En protein revealed a marked and uniform expansion of its expression in shdBili embryos (Fig. 2E–I). Wild type embryos at stage 12 (germ band retraction) and the ventral epidermis at stage 14 (onset of head involution and dorsal closure) have approximately two rows of cells expressing En (Fig. 2, F and G). These regions were expanded to approximately four rows of cells in the presence of shdBili (Fig. 2, H and I). These data further support the conclusion that dBili is an inhibitor of Wg signaling during *Drosophila* embryonic development.

### Bili is conserved in vertebrates and negatively regulates Wnt/β-catenin signaling in zebrafish

**Figure 3:**
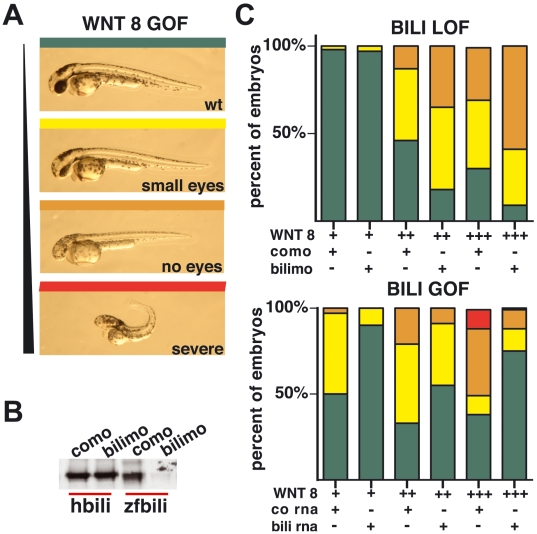
Bili's effect on Wnt signaling is conserved in zebrafish. (A) Wnt8 overexpression phenotypes with increasing severity. Embryos were scored as wt (green), small eyes (yellow), no eyes (orange) or severe (red). (B) Morpholino effect on in vitro expression of human Bili (hBili) or zfBili protein (control (como) or zfBili morpholino (bilimo)). (C) In the top panel, the effect of bilimo (loss of function) on Wnt8 GOF phenotype compared to como, is shown (n = 73 low Wnt dose, n = 147 medium Wnt dose, n = 57 high Wnt dose). Bili GOF (bili rna) rescues Wnt8 overexpression phenotype (control *Renilla* (co rna)). (n = 125 per dose of Wnt8) (bottom panel).

The Wg signaling pathway is highly conserved throughout evolution. Therefore, we next asked if Bili negatively regulates Wnt signaling in vertebrates. To this end, we asked if Bili function is conserved in zebrafish. Ectopic activation of Wnt/β-catenin signaling in early zebrafish development causes dose-dependent anterior truncations and mesodermal defects (Fig. 3A) [17]. If zfBili functions as a negative regulator of Wnt/β-catenin signaling during zebrafish development, then silencing zfBili expression should exacerbate these phenotypes. Overexpression of Wnt8 results in anterior truncation phenotypes of varying severity (Fig. 3A). Silencing Bili with either of two non-overlapping antisense morpholinos targeting zfBili (bilimo or bilimo2) shifted the Wnt8 induced phenotypes toward increasing severity (Fig. 3C, top panel, Fig. S4E). As controls, we first confirmed that zfBili is expressed throughout early zebrafish development (Fig. S4A–D) and second verified that bilimo, but not a control morpholino (como), repressed translation of zfBili (Fig. 3B).

We next investigated the effect of zfBili gain-of-function on zebrafish development. Embryos injected with zfBili RNA, but not control RNA, displayed a slight expansion of anterior structures, which is consistent with diminished Wnt/β-catenin signaling (data not shown) [18]. Importantly, zfBili overexpression rescued Wnt8 gain-of-function phenotypes (Fig. 3C, bottom). Together, these results demonstrate that Bili is an evolutionarily conserved negative regulator of Wnt/β-catenin signaling.

### Bili negatively regulates Wnt/β-catenin signaling in human cultured cells

**Figure 4:**
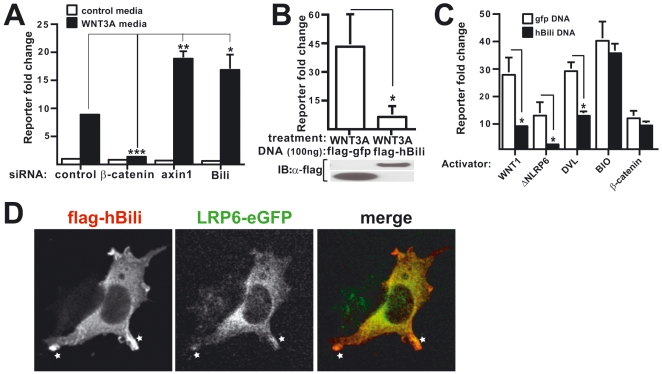
Bili inhibits Wnt/β-catenin transcriptional reporters in human cell-lines. (A) Pretreatment with Bili or Axin1 siRNA leads to a 2-fold greater activation of a β-catenin responsive reporter after treatment with WNT3A conditioned media (black bars) in RKO-BAR cells compared to control siRNA. β-catenin (β-cat) siRNA ablated Wnt 3A mediated reporter activation. Error bars represent STDEV. (B) hBili overexpression inhibits Wnt3A mediated β-catenin responsive reporter activation in HEK293T cells. (C) hBili overexpression inhibits WNT1, ΔNLRP6, and DVl, but not BIO, or β-catenin mediated β-catenin responsive reporter activation in HEK293T cells. HEK293T cells stably expressing a β-catenin responsive luciferase reporter were transfected or treated with WNT1, ΔNLRP6, DVL, BIO, or β-catenin along with GFP or Bili. Error bars represent STDEV. (D) Bili colocalizes with LRP6. HEK293T cells were transfected with flag-hBili and hLRP6-eGFP and imaged. (*p<0.05, **p<0.005, ***p<0.0005)

We next tested whether Bili regulates Wnt signaling in human cells. We performed siRNA knockdown or cDNA overexpression of hBili in RKO colorectal carcinoma cells or human embryonic kidney (HEK293T) cells expressing a β-catenin responsive luciferase reporter and *Renilla* luciferase normalization control. Activation of the reporter in RKO cells following treatment with WNT3A conditioned media was enhanced by transfection of either hBili or *AXIN1* siRNA and repressed by β-catenin siRNA (Fig. 4A). Similarly, activation of the reporter in HEK293T cells by transfection of *WNT1*, a pathway activator, was enhanced by hBili siRNA and repressed by β-catenin siRNA (Fig. S5A). Consistent with these loss-of-function studies, overexpression of hBili in HEK293T cells inhibited WNT3A-mediated activation of the reporter (Fig. 4B).

A series of additional controls confirm these results and the conclusion that Bili is a negative regulator of the Wnt/β-catenin pathway in human cells. First, hBili siRNA effectively reduced both hBili mRNA and tagged protein levels. By qRT-PCR, hBili siRNA depleted hBili transcript levels by 50% (Fig. S5B), and reduced levels of overexpressed hBili protein (Fig. S5C). Second, four additional independent siRNAs designed against hBili had a similar or greater effect on Wnt-mediated activation of the β-catenin responsive reporter (Fig. S5D and E and data not shown) ruling out the formal possibility that the activity of the first siRNA was due to off-target effects. Third, hBili siRNA synergized with WNT3A conditioned media to induce transcriptional upregulation of the endogenous β-catenin target gene, *AXIN2* (Fig. S5F). This effect on expression of an endogenous β-catenin target gene validates the conclusions of the synthetic reporters. Fourth, knockdown or overexpression of hBili had no effect on control β-catenin unresponsive reporters (Fig. S6A andB) or a CREB reporter that was activated with forskolin (Fig. S6C). These data collectively support the conclusion that Bili is a conserved inhibitor of Wnt/β-catenin signaling in diverse species.

### Bili functions upstream of β-catenin stabilization and associates with LRP6

We next carried out epistasis experiments in HEK293T cells expressing a β-catenin responsive luciferase reporter and *Renilla* luciferase normalization control. hBili or GFP were overexpressed in the presence of BIO (6-bromoindirubin-3′-oxime), a GSK3 inhibitor [19], or co-expressed with other pathway activators including WNT1, ΔNLRP6, Dishevelled (DVL) or β-catenin. Bili gain-of-function reduced reporter activation by WNT1, ΔNLRP6, and DVL (Fig. 4C). In contrast, hBili overexpression did not affect reporter activation by overexpression of β-catenin or BIO treatment (Fig. 4C). These results place the function of hBili between the Wnt receptor complex and the β-catenin degradation complex. Consistent with these epistasis studies, and the observed membrane associated localization of other FERM domain containing proteins, we found that dBili localizes in a concentric ring, adjacent to Arm protein at the plasma membrane in fly cells (Fig. S3E and S3E').

**Figure 5:**
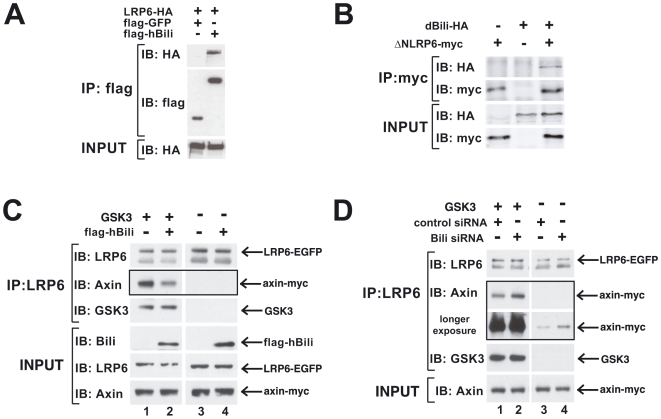
Bili associates with LRP6 and attenuates Axin recruitment to LRP6 in HEK293T cells. (A) hBili interacts with LRP6. HA-LRP6 was cotransfected with flag-GFP or flag-hBili in HEK293T cells. Cell lysates were immunoprecipitated with anti-flag antibody and immunoblotted with anti-HA antibody. (B) dBili interacts with LRP6. dBili-HA and ΔNLrp6-myc were cotransfected in S2R+ cells. Cell lysates were immunoprecipitated with anti-myc antibody and immunoblotted with anti-HA antibody. (C) Overexpression of Bili attenuates GSK3 induced AXIN/LRP6 association. HEK293T cells stably expressing LRP6-eGFP were cotransfected with AXIN-myc, GSK3B and Bili-flag or vector control. Cell lysates were immunoprecipitated with anti-eGFP antibody and immunoblotted with anti-myc. Data shown are representative of three independent experiments. (D) Bili knockdown enhances GSK3B induced AXIN/LRP6 association. HEK293T cells stably expressing LRP6-eGFP were transfected with Bili siRNA or control siRNA. 48 hours later BilisiRNA or control siRNA treated cells were transfected with AXIN-myc and GSKB. Cell lysates were immunoprecipitated with anti-eGFP antibody and immunoblotted with anti-myc. Data shown are representative of three independent experiments.

Based on these results we predicted that Bili might regulate signaling from the Wnt co-receptors LRP5/6 or Frizzled. In direct support of this prediction, tagged hBili colocalizes with tagged LRP6 as detected by immunocytochemistry (ICC) (Fig. 4D). Furthermore, hBili-FLAG pulls down tagged-LRP6 in co-immunoprecipitation (co-IP) assays (Fig. 5A). The association of Bili and LRP6 was also confirmed in Drosophila S2R+ cells where immunoprecipitated tagged-ΔNLRP6 pulled down tagged-dBili (Fig. 5B). Collectively, these data suggest that Bili functions upstream of β-catenin stabilization and associates with the LRP6 receptor complex.

### Bili antagonizes the recruitment of Axin to LRP6

During Wnt signaling, LRP6 is phosphorylated in a Wnt-dependent manner by CSNK1G1 and GSK3B, which then facilitates the binding of AXIN to LRP6 [4], [5]. Consistent with this model, overexpression of GSK3B consistently and robustly mimics the ability of WNT to induce the binding of AXIN to LRP6 ([20], [21]. Our observations that Bili negatively regulates Wnt/β-catenin signaling upstream of the destruction complex and associates with LRP6 lead us to hypothesize that Bili may regulate the recruitment of AXIN to LRP6.

To address this hypothesis we employed co-immunoprecipitation to monitor the association of AXIN and LRP6 following gain-of-function (Fig. 5C) or loss-of-function (Fig. 5D) of Bili. HEK293T cells stably expressing LRP6-EGFP were transiently transfected with AXIN-MYC. As expected LRP6 co-immunoprecipitated AXIN in the presence of overexpressed GSK3B (Fig. 5C & 5D, lane 1 compared to lane 3). This interaction of LRP6 and AXIN was attenuated approximately 2-fold in the presence of overexpressed hBili (Fig. 5C, lane 2 compared to lane 1) and enhanced more than 2-fold when hBili levels were knocked down (Figure 5D, lane 2 compared to lane 1). Furthermore, even in the absence of GSK3B, knockdown of hBili enhanced the association of AXIN and LRP6 approximately 4-fold (Fig. 5D, lane 4 compared to lane 3). Together these data suggest that Bili negatively regulates the recruitment of AXIN to LRP6 thereby providing a mechanism for how Bili negatively regulates Wnt/β-catenin signaling.

## Discussion

Many aspects of Wnt/β-catenin signal transduction remain poorly understood. One aspect in particular is the transduction events occurring at the plasma membrane following Wnt activation of the Frizzled/LRP6 coreceptor complex. Studies over the last several years have shed light on these events by uncovering several kinases that phosphorylate LRP6 resulting in its recruitment of AXIN [4], [5]. As AXIN recruitment to LRP6 appears to be a key step in the propagation of a WNT signal, it is likely a hotspot for pathway regulation. Our identification of Bili as a negative regulator of Wnt/β-catenin signaling and inhibitor of AXIN recruitment to LRP6 is the first report of negative regulation at this step in pathway transduction. The specific details of how Bili affects AXIN recruitment will be the focus of future studies. One proposed mechanism is that Bili sterically hinders the recruitment of the kinases required for LRP6 phosphorylation and subsequent AXIN recruitment. A second possibility is that Bili inhibits the formation of LRP6 aggregates or ‘signalosomes’ which have been shown to be the LRP6 population associated with AXIN (Bilic et al. 2007).

We propose that inhibitors of the Wnt/β-catenin pathway fall into two groups: ‘constitutive inhibitors’ that keep basal levels of β-catenin low, such as members of the degradation complex; and ‘inducible inhibitors’ that act outside of the degradation complex and may function only when the pathway has been activated. The ‘constitutive inhibitors’ would include APC and AXIN, while ‘inducible inhibitors’ would include NKD and Bili. Consistent with this categorization, NKD and Bili both antagonize a WNT signal between receptor activation and the degradation machinery as well as having similar phenotypes in both flies [22] and fish [23]. In concert, constitutive and inducible inhibitors allow for rapid pathway activation in response to WNT ligand and tight regulation of the strength and duration of the WNT signal. Continued characterization and identification of both constitutive and inducible inhibitors is key to our understanding of the regulation of Wnt/β-catenin signaling in development and disease.

## Materials and Methods

### Fly screen

The screen was performed as previously described [10].

### Reporter assays

β-catenin reporter assays were carried out in the 24-well or 48-well plate format in HEK293T cells transiently expressing SuperTOPFLASH (1–5 ng) or BAR (β-catenin Activated Reporter) and *Renilla* luciferase (10–40 ng) for normalization. FOPFlash and fuBAR, which contain mutated response elements and do not respond to WNT/β-catenin signaling, were used as control reporters. We used RKO cells that stably express BAR and *Renilla* [24]. In LOF studies, cells were seeded to 30% confluency before siRNAs were transfected using Lipofectamine RNAimax (invitrogen) at a concentration of 20 µM. 24 h later the cells were treated with L-cell media or WNT3A media (or transfected with *WNT1* (0.1 ng) and TOPFLASH (1 ng)/*Renilla* luciferase(10 ng)). Lysis and luminescence (using Promega dual-luciferase assay kit) was carried out 24 h after WNT treatment. For GOF experiments, cells were seeded to 50% confluency after which cDNA (GFP (50–100 ng) or Bili-FLAG (25–100 ng), TOPFLASH, *Renilla* luciferase, *WNT1* (0.1 ng), ΔNLRP6 (5 ng), DVL (5 ng), β-catenin (1 ng) was transfected. BIO (0.5 µM) was also added to the cells at this point. Cells were lysed and luminescence measured 24 h hours later. Bili siRNAs were purchased from Ambion; siRNA ID# 332724 that targeted Exon 8 (sense 5′ggcgugcacgucaucgauatt3′, antisense 5′uaucgaugacgugcacgcctt3′) and siRNA ID# 45515 that targeted Exon 7, 8. Control siRNA and Axin 1 & 2 siRNAs have been described [24].

### *Drosophila* in situ hybridization and immuno-staining

In situ hybridization and immuno-staining in the fly embryos and larval imaginal discs were performed using standard protocols described in the laboratory manual “*Drosophila Protocols*” by William Sullivan et al., CHSL press.

### qRT-PCR in *Drosophila* embryos

RNA was isolated from Drosophila embryos using Trizol and was further purified using the RNA “clean-up” protocol with the Qiagen RNAeasy kit. This was followed by reverse transcription (Applied Biosystems - High Capacity cDNA Reverse Transcription Kit) and quantitative PCR (SYBR green) using Applied Biosystems 7300 Real-Time PCR Machine. PCR primers used were as follows: Actin: Act Forward 5′ – GAA GAT CTC CCA ATC GGC GAA CAA TTC ATA CC – 3′; Act Reverse 5′ - GAA GAT CTT TGA ACG CGA CTT GAG AGC GG – 3′; Armadillo: Arm Forward 5′ – CAA CGT TCC TCG ATA GCC AC – 3′; Arm Reverse 5′ – CGA ATG AGT GCC CGG TTG TG – 3′; C11848/dBili: CG11848 Forward 5′ – CTT GGC TGT GGC TAC CGA TC - 3′; CG11848 Reverse 5′ – GCG CCA TAG AAT GGC AGA GC – 3′.

### Constructs

MGC clone for human Bili (MHS1010-9204707) was purchased (Open biosystems). Bili was sub-cloned into pCS2+ using the EcoR1 and Xho1 sites. Forward primer sequence 5′ GTAGTAAAGCTTGCCACCATGGCCCTGAGGATGGACGGG 3′ and reverse primer sequence 5′ TACTACGTCGACTCAGCCCTGCTCCAGGCTGT 3′. Zebrafish Bili was cloned out of a mixed embryonic library (6 hpf-24 hpf) using forward primer 5′ TCTCCAGCTCAGGATTTGTTGGTG 3′ and reverse primer 5′ TACTACCTCGAGTCAACTTTCAGTCAC 3′ based on an ENSEMBLE BLAST prediction using Bili. Since the ATG was missing, a 5′ RACE kit (Invitrogen) was used with a nested primer 5′ GCCACTGACGACACAGCTTGTAA 3′ that yielded the full-length clone.

### Co-immunoprecipitation

HEK293T cells were made to stably express LRP6-EGFP by transfecting pCS2+LRP-EGFP and pPUR (Clontech) into cells and treating with 2 mg/ml of puroMYCin. Stable cells were seeded into 6-well plates at 50% confluency and transfected with *AXIN*-MYC (300 ng), *GSK3B* (50 ng) and Bili-FLAG (200 ng). Cells were lysed the next day in a buffer containing 1% Triton-X, 50 mM Tris-HCL pH 7, 150 mM NaCl, protease and phosphatase inhibitors. LRP6-EGFP was immunoprecipitated using polyclonal GFP and protein G beads for 2 hours. The beads were washed three times with the lysis buffer that contained 0.6 M NaCl instead of 150 mM NaCl. Proteins that bound were eluted using SDS gel loading buffer and western blot analysis was used to detect GFP, MYC, FLAG and GSK3B. Quantification was done using NIH image.

Drosophila S2R+ cells were used for coIP. S2R+ cells were co-transfected with HA-tagged dBili and Myc-tagged,ΔNLRP6 IP was performed with Rabbit anti-Myc antibody (1∶50, Upstate-Cell Signaling Solutions, Cat # 06-549) and IB with both anti-Myc (1∶500) and anti-HA (1∶1000, Roche Cat # 1 867 423). Standard IP protocol was used with mild lysis buffer and Protein A/G sepharose.

### Zebrafish in situ hybridization and microinjection

*In situ* hybridization and microinjections were carried out as previously described on zebrafish online resource, ZFIN. ATG blocking morpholino sequences (Gene Tools) GGAAGTCGCCATCATCTCCCTCCAT (bilimo) and GAGACACTCTCCTTCGATTCAGAAG (bilimo2). A 10 µM stock was diluted to 1.5 µM. bilimo, bilimo2, or control morpholino at this concentration were then coinjected into 1-cell embryos at three doses of *wnt8* RNA (stock concentrations 2 ng/µl, 4 ng/µl and 8 ng/µl). The same wnt doses were used for zfBili overexpression coinjection experiments with *Renilla* as a control at 50 ng/µl stock concentration. Injection drop size 0.5–1 nl.

### QT-RTPCR

RNA was isolated following treatment using Qiagen RNAeasy kit. This was followed by reverse transcription (Invitrogen ThermoScript or SuperScript) and quantitative PCR (sybr green) using Roche Light cycler 2.0. PCR primers Bili forward 5′CCAAGCAGGCCGAACT3′, Bili reverse 5′CCTTGCCGTCCTCCACGTA3′, GAPDH forward 5′CCA CCC ATG GCA AAT TCC ATG GCA3′, GAPDH reverse 5′TCTAGACGGCAGGTCAGGTCCACC3′. Axin 2 forward 5′CTCCCCACCTTGAATGAAGA3′ Axin 2 reverse 5′ TGGCTGGTGCAAAGACATAG3′

#### Gene accession numbers

NM_143085 (Drosophila), XM_682750.2 (Zebrafish), NM_031904 (Human).

## Supporting Information

Table S1 Table showing dsRNA targeting eighteen genes with known human orthologs that increased reporter activation two-fold or greater.
(0.01 MB DOC)

Figure S1 (A) Sequence alignment of Bili protein shows it is well conserved phylogenically. (B) Human Multi Tissue Northern blot show mRNA expression. (C) Illustration of Bili protein structure and domains.
(2.14 MB EPS)

Figure S2 (A) qRT-PCR analysis of dBili knockdown. Expression of short-hairpin (shRNA) dbili (#3M) under the control of prd-GAL4 in the Drosophila embryo results in >75% knockdown in message level (Red bar) as compared to prd-GAL4 control embryos (Blue bar). As a control, Armadillo mRNA was not affected in embryos expressing dBili shRNA. (B) Embryos expressing dBili shRNA under the control of even skipped stripe 3/7-GAL4: These embryos display a partial lack/disruption of the 2nd or 3rd denticle belt which coincides with the eve-stripe 3 expression. This phenotype is consistent with a localized increase in Wingless signaling activity in the region around eve-stripe 3. The same however was not observed for stripe 7 (posterior end of the embryo), perhaps due to differential expression of GAL4 in stripe 3 versus stripe 7.
(3.11 MB EPS)

Figure S3 (A) Knockdown of dBili with a second dsRNA, ds11848_2, revealed only ∼30% knockdown of endogenous message compared to the original dsRNA (DRSC1.0-CG11848) which robustly knocked down message levels by >50%. Primer sequence used for the generation of ds11848_2 PCR is as follows: Forward primer: 5′-GTAATACGACTCACTATAGGGAGA GAAGATACAAGTGAGGCATTC-3′ and Reverse primer: 5′GTAATACGACTCACTATAGGGAGAGGCAAATAAAATATCTGATGGGTGCGTGG-3′ (B) Knockdown of dBili with ds11848_2 displayed a modest increase (∼35–40%) in dTF12 (Wg-reporter) activity. (C & D) Expression of dBili-HA strongly inhibits dTF12-reporter activity when the pathway is activated by Wg overexpression. The inhibitory effect of dBili is comparable to that of Axin expression in both S2R+ (C) and clone8 (D) cells. (E) dBili protein (in GREEN) is localized in a concentric ring, just inside and abutting Arm protein (in RED) at the membrane of cells in the drosophila embryo. (E') Magnitifed view of boxed region in panel E.
(5.47 MB EPS)

Figure S4 The zebrafish homolog of Bili (zfBili) is expressed during embryogenesis. In situ hybridization using a sense (left column) and antisense probes (right column) were used to detect mRNA. (A) zfBili is expressed maternally as it is detected in the animal pole 4 h post-fertilization. (B) zfBili continues to be expressed ubiquitously at 50% epiboly. (C) 24 hpf zfBili remains ubiquitous but shows specific staining in the otic vesicle (arrow head). (D) Weak Bili expression in the tail at 36 hpf. (E) A second morpholino targeting zfBili (bilimo2) but not a control morpholino (como) enhances the Wnt8 overexpression phenotype (n = 50 for each condition). Embryos were scored as wt (green), small eyes (yellow), no eyes (orange) or severe (red). Data shown is representative from four independent experiments.
(10.82 MB EPS)

Figure S5 Bili negatively regulates Wnt/{capital β-catenin signaling in mammalian cells. (A) siRNA mediated knockdown of hBili enhances Wnt1 mediated induction of a β-catenin responsive reporter. HEK293T cells were transfected with siRNA targeting control, β-catenin, or hBili. Cells were then transfected with WNT1 cDNA and a β-catenin responsive reporter and assayed the following day. (B) hBili siRNA effectively knocked down hBili transcripts in HEK293T as measured by qRT-PCR. (C) hBili siRNA effectively decreased the expression of a hBili-Venus fusion protein as assayed by fluorescence measurement. hBili siRNA had no effect on Venus expression. (D) A second siRNA (Bili #2) targeting hBili enhanced WNT1 mediated activation of a β-catenin responsive reporter. (E) Bili #2 siRNA also effectively decrease expression of a hBili-Venus fusion protein with no effect on Venus alone. (F) hBili knockdown in HEK293T cells with hBili siRNA enhanced the WNT3A mediated transcriptional induction of endogenous AXIN2 transcripts as assayed by qRT-PCR. Error bars represent STDEV (*p<0.05, **p<0.005, ***p<0.0005).
(1.48 MB EPS)

Figure S6 Bili does not affect control reporters or CREB responsive reporters. (A) HEK293T cells were transfected with FUBAR, a control reporter not responsive to Wnt/β-catenin signaling, renilla luciferase normalization, and control or hBili siRNA. FUBAR was not responsive to WNT3A conditioned media (right two bars) and hBili siRNA had no effect. (B) HEK293T cells were transfected with FOPFlash, another control reporter not responsive to Wnt/β-catenin signaling, renilla luciferase normalization, and GFP cDNA or increasing doses of hBili cDNA or control siRNA or hBili siRNA. Neither hBili cDNA control nor hBili siRNA had an effect on the reporter. (C) hBili siRNA HEK293T cells stably expressing a CREB responsive reporter were transfected with renilla luciferase normalization, and control, hBili, or hBili#2 siRNA and treated with DMSO, 1 uM forskolin, or 10 uM forskolin. Error bars represent STDEV.
(1.14 MB EPS)

## References

[1] Clevers H (2006). Wnt/beta-catenin signaling in development and disease.. Cell.

[2] Kimelman D, Xu W (2006). beta-catenin destruction complex: insights and questions from a structural perspective.. Oncogene.

[3] Bilic J, Huang YL, Davidson G, Zimmermann T, Cruciat CM (2007). Wnt induces LRP6 signalosomes and promotes dishevelled-dependent LRP6 phosphorylation.. Science.

[4] Zeng X, Tamai K, Doble B, Li S, Huang H (2005). A dual-kinase mechanism for Wnt co-receptor phosphorylation and activation.. Nature.

[5] Davidson G, Wu W, Shen J, Bilic J, Fenger U (2005). Casein kinase 1 gamma couples Wnt receptor activation to cytoplasmic signal transduction.. Nature.

[6] Lee E, Salic A, Kruger R, Heinrich R, Kirschner MW (2003). The roles of APC and Axin derived from experimental and theoretical analysis of the Wnt pathway.. PLoS Biol.

[7] He X, Semenov M, Tamai K, Zeng X (2004). LDL receptor-related proteins 5 and 6 in Wnt/beta-catenin signaling: arrows point the way.. Development.

[8] Bejsovec A (2006). Flying at the head of the pack: Wnt biology in Drosophila.. Oncogene.

[9] Friedman A, Perrimon N (2007). Genetic screening for signal transduction in the era of network biology.. Cell.

[10] DasGupta R, Kaykas A, Moon RT, Perrimon N (2005). Functional genomic analysis of the Wnt-wingless signaling pathway.. Science.

[11] Liu G, Bafico A, Harris VK, Aaronson SA (2003). A novel mechanism for Wnt activation of canonical signaling through the LRP6 receptor.. Mol Cell Biol.

[12] Louvet-Vallee S (2000). ERM proteins: from cellular architecture to cell signaling.. Biol Cell.

[13] Hendriksen J, Fagotto F, van der Velde H, van Schie M, Noordermeer J (2005). RanBP3 enhances nuclear export of active (beta)-catenin independently of CRM1.. J Cell Biol.

[14] Bejsovec A, Martinez Arias A (1991). Roles of wingless in patterning the larval epidermis of Drosophila.. Development.

[15] Bejsovec A, Wieschaus E (1993). Segment polarity gene interactions modulate epidermal patterning in Drosophila embryos.. Development.

[16] Wodarz A, Nusse R (1998). Mechanisms of Wnt signaling in development.. Annu Rev Cell Dev Biol.

[17] Kelly GM, Greenstein P, Erezyilmaz DF, Moon RT (1995). Zebrafish wnt8 and wnt8b share a common activity but are involved in distinct developmental pathways.. Development.

[18] Erter CE, Wilm TP, Basler N, Wright CV, Solnica-Krezel L (2001). Wnt8 is required in lateral mesendodermal precursors for neural posteriorization in vivo.. Development.

[19] Meijer L, Flajolet M, Greengard P (2004). Pharmacological inhibitors of glycogen synthase kinase 3.. Trends Pharmacol Sci.

[20] Mao J, Wang J, Liu B, Pan W, Farr GH (2001). Low-density lipoprotein receptor-related protein-5 binds to Axin and regulates the canonical Wnt signaling pathway.. Mol Cell.

[21] Yamamoto H, Komekado H, Kikuchi A (2006). Caveolin is necessary for Wnt-3a-dependent internalization of LRP6 and accumulation of beta-catenin.. Dev Cell.

[22] Zeng W, Wharton KA, Mack JA, Wang K, Gadbaw M (2000). naked cuticle encodes an inducible antagonist of Wnt signalling.. Nature.

[23] Wharton KA, Zimmermann G, Rousset R, Scott MP (2001). Vertebrate proteins related to Drosophila Naked Cuticle bind Dishevelled and antagonize Wnt signaling.. Dev Biol.

[24] Major MB, Camp ND, Berndt JD, Yi X, Goldenberg SJ (2007). Wilms tumor suppressor WTX negatively regulates WNT/beta-catenin signaling.. Science.

