# Supplementary material for: Bili Inhibits Wnt/β-Catenin Signaling by Regulating the Recruitment of Axin to LRP6
Source: PLoS One. 2009 Jul 2;4(7):e6129. doi: 10.1371/journal.pone.0006129 (PMC2701632; doi:10.1371/journal.pone.0006129)
Supplement: Table S1 — Table showing dsRNA targeting eighteen genes with known human orthologs that increased reporter activation two-fold or greater. (0.01 MB DOC) [file pone.0006129.s001.doc]

**Supplemental Table**

| **Lrp (#1)** | **P'type in 1˚ Scrn** | **∆NLrp6** | **fold change** | **no induc** | **fold change** | **predicted/determined localization** |
| --- | --- | --- | --- | --- | --- | --- |
| CG1135 | *increased* | 334 | 3.5 | 320 | 3.2 | nucleus |
| CG18041 | *increased* | 302 | 3.2 | 339 | 3.4 | unknown |
| Bx42 | *increased* | 290 | 3.0 | 267 | 2.7 | nucleus |
| bel | *increased* | 281 | 3.0 | 323 | 3.2 | nucleus/cytoplasm |
| CG31992 | *increased* | 257 | 2.7 | 136 | 1.4 | cytoplasm |
| Trn | *increased* | 243 | 2.6 | 248 | 2.5 | nuclear membrane/cytoplasm |
| CG6686 | *increased* | 242 | 2.5 | 213 | 2.1 | nucleus/cytoplasm |
| **ran** | ***increased*** | **229** | **2.4** | **309** | **3.1** | **cytoplasm** |
| CG1017 | *increased* | 229 | 2.4 | 160 | 1.6 | extracellular |
| CG15432 | *increased* | 219 | 2.3 | 757 | 7.6 | unknown |
| **CG11848** | ***increased*** | **218** | **2.3** | **144** | **1.4** | **plasma membrane/cytoskeleton** |
| CG16903 | *increased* | 218 | 2.3 | 139 | 1.4 | nucleus/cytoplasm |
| Pp1-13C | *increased* | 218 | 2.3 | 651 | 6.5 | nucleus/cytoplasm |
| CG8435 | *increased* | 202 | 2.1 | 213 | 2.1 | unknown |
| ash1 | *increased* | 199 | 2.1 | 93 | 0.9 | nucleus |
| Fs(2)Ket | *increased* | 199 | 2.1 | 157 | 1.6 | nucleus/cytoplasm |
| Hsc70-3 | *increased* | 198 | 2.1 | 136 | 1.4 | ER, plasma membrane, nucleolus, cytoplasm |
| enok | *increased* | 198 | 2.1 | 171 | 1.7 | nucleus |
